# Novel and disappearing climates in the global surface ocean from 1800 to 2100

**DOI:** 10.1038/s41598-021-94872-4

**Published:** 2021-08-26

**Authors:** Katie E. Lotterhos, Áki J. Láruson, Li-Qing Jiang

**Affiliations:** 1 Northeastern University Marine Science Center, 430 Nahant Rd, Nahant, MA 01908 USA; 2 Department of Natural Resources, Cornell University, Ithaca, NY 14850 USA; 3 Earth System Science Interdisciplinary Center, University of Maryland, College Park, MD 20740 USA; 4 National Centers for Environmental Information, National Oceanic and Atmospheric Administration, Silver Spring, MD 20910 USA

**Keywords:** Climate and Earth system modelling, Environmental health, Climate-change ecology

## Abstract

Marine ecosystems are experiencing unprecedented warming and acidification caused by anthropogenic carbon dioxide. For the global sea surface, we quantified the degree that present climates are disappearing and novel climates (without recent analogs) are emerging, spanning from 1800 through different emission scenarios to 2100. We quantified the sea surface environment based on model estimates of carbonate chemistry and temperature. Between 1800 and 2000, no gridpoints on the ocean surface were estimated to have experienced an extreme degree of global disappearance or novelty. In other words, the majority of environmental shifts since 1800 were not novel, which is consistent with evidence that marine species have been able to track shifting environments via dispersal. However, between 2000 and 2100 under Representative Concentrations Pathway (RCP) 4.5 and 8.5 projections, 10–82% of the surface ocean is estimated to experience an extreme degree of global novelty. Additionally, 35–95% of the surface ocean is estimated to experience an extreme degree of global disappearance. These upward estimates of climate novelty and disappearance are larger than those predicted for terrestrial systems. Without mitigation, many species will face rapidly disappearing or novel climates that cannot be outpaced by dispersal and may require evolutionary adaptation to keep pace.

## Introduction

Marine ecosystems worldwide are being threatened by an anticipated temperature increase of 1–3 °C^1^ and a pH drop of 0.3–0.5 units (an acidity increase of greater than 100%)^2,3^ over the next century due to the uptake of atmospheric carbon dioxide (CO_2_)^4–6^. The rates of change in atmospheric CO_2_ over the past century are two-to-three orders of magnitude higher than most of the changes seen in the past 420,000 to 300 million years, suggesting that this challenge may be without precedent for many extant species^4–6^. This rapid rate of environmental change means that by the end of the twenty-first century, large portions of the Earth’s ocean could experience climates not found at present (“novel climates”), and some twentieth century climates may disappear^7–9^.

Despite evidence that some marine species may be able to keep pace with climate change through distribution shifts because of high dispersal potential^10–12^, range shifts no longer become a viable strategy if globally the climate shifts beyond what they can tolerate. Thus, novel climates with no analog in recent evolutionary history may leave species in an “adapt or die” scenario^13^. In addition, novel climates may cause a reshuffling of communities including novel species associations, community disaggregation, new communities, extinction, and other unexpected ecological surprises^7,8,14^.

Recently for the global ocean, others have estimated the year that single climate variables (e.g., pH, SST, oxygen) are projected to emerge beyond a historical baseline for a particular location or marine reserve^15–17^. While these kinds of analyses are important, they did not give insight into where novel environmental stresses not recently experienced anywhere on Earth may emerge, nor where historical climates may disappear relative to a global baseline. In addition, these previous studies did not quantify the degree of climate novelty or disappearance in the global ocean since pre-industrial times.

Our study fills these gaps by quantifying the degree of global climate novelty or disappearance for the ocean sea surface, based on the dissimilarity between the multivariate climate normal at a focal geographic location and its nearest analog in the climate normals from the global climate baseline data (Table 1, definitions). We use reconstructed pre-industrial environments and climate change scenarios to map risk of current and future novel and disappearing environments for the global sea surface and discuss their potential ecological impacts. The degree of global novelty is calculated by comparing a *later* climate normal for each surface ocean gridpoint to a baseline of climate normals for all surface ocean gridpoints in the same hemisphere (N or S) from an *earlier* time. Gridpoints with a high degree of global novelty are those whose future climate projection lies outside of the present-day climate envelope for that hemisphere. In contrast, the degree of global disappearance is calculated by comparing each gridpoint at an *earlier* time to a baseline of climate normals for all surface ocean gridpoints in the same hemisphere from a *later* time (Table 1, definitions). Gridpoints with a high degree of global disappearance are those whose present-day climate lies outside of the future-projected climate envelope for that hemisphere.

**Table 1:** Definitions for the terms used in this study in alphabetical order.

Term	Definition
Climate normal	In this study, 40-year means of each climate variable obtained from the model for a single ocean gridpoint
Degree of global novelty (*σ*_D-Novelty_)	Calculated by comparing the climate normal for each ocean gridpoint at a *later* time to a pool of climate normals from the global climate baseline data from an *earlier* time. Mathematically, *σ*_D-Novelty_ is an estimate of the dissimilarity between the *later* climate normal for a focal geographic location and its nearest neighbor in the global climate baseline data from an *earlier* pool of climate normals^30^
Degree of global disappearance (*σ*_D-Disappearance_)	Calculated by comparing the climate normal for each ocean gridpoint at an *earlier* time to a pool of climate normals from the global climate baseline data from a *later* time. Mathematically, *σ*_D-Disappearance_ is an estimate of the dissimilarity between an *earlier* climate normal for a focal geographic location and its nearest neighbor in the global climate baseline data from a *later* pool of climate normals^30^
Degree of global novelty/disappearance—moderate^30^	2-4*σ*_D_ degree of sigma dissimilarity; corresponds to the 95th percentile of the global climate baseline data
Degree of global novelty/disappearance—extreme^30^	Greater than 4*σ*_D_ degree of sigma dissimilarity; corresponds to the 99.994th percentile of the global climate baseline data
Focal station or focal geographic location	The location for which the degree of climate novelty or disappearance is being calculated. In this study, the focal stations are individual ocean gridpoints
Global climate baseline data	Includes climate normals for the sea surface from widespread geographic locations in the hemisphere of the focal station (e.g., northern or southern hemisphere) at a specific point in time. For the degree of global novelty, the baseline consists of climate normals from an earlier time point than the focal station. For the degree of global disappearance, the baseline consists of climate normals from a later time point than the focal station
Interannual climate variability (ICV)^30^	The flucuations in climate observed at the focal station, which is used to standardize *M*_*D*_ into *σ*_D_. In this study, ICV for each focal station included all model observations between 1965 and 2004
Mahalanobis distance (*M*_*D*_)^30^	The multivariate distance between a single gridpoint at one point in time and its closest analog (nearest neighbor) in the global climate baseline data from another time point
Nearest neighbor	In principal components space (following standardization by ICV), the nearest neighbor is the geographical location in the global climate baseline data whose climate normal (at one point in time) is most similar to the climate normal at the focal station at a different point in time (e.g., closest analog). For the degree of global novelty, the nearest neighbor is the geographical location in the global data whose climate at an *earlier* time is most similar to that of the climate at the focal station at a *later* time. For the degree of global disappearance, the nearest neighbor is the geographical location in the global data whose climate at a *later* time is most similar to that of the climate at the focal station at an *earlier* time
Ocean climate	In this study, ocean climate is quantified by seasonal temperature, pH, and the saturation state of aragonite (a form of calcium carbonate form found in corals, bivalves, and many other marine organisms)
Sigma dissimilarity (*σ*_D_)^30^	The transformation of *M*_*D*_ into a standardized metric that can be interpreted as the number of standard deviations of interannual climate variability (ICV) at the focal station

Unlike on land, where the climate is traditionally described by temperature and precipitation, here we consider ocean climate to be described by temperature and carbonate chemistry (Table 1, definitions). Carbonate chemistry is an important aspect of ocean climate because it describes the availability of biologically important carbon ions (CO_3_^2−^) that many marine fauna use to make shells or bone. We calculated the degree of global novelty or disappearance based on seasonal temperature, pH, and the saturation state of aragonite: a form of calcium carbonate form found in corals, bivalves, and many other marine organisms^18–20^. These three variables describe different aspects of the ocean climate. For instance, temperature is known to be an important driver of biodiversity in the marine environment^21^ through its influence on the biochemical kinetics of metabolism^22^, thermal tolerance limits^10^, and the sensitivity of corals to warming^23^. Saturation state and pH are interrelated and both decrease with increasing CO_2_, but have distinct effects on organisms. Declines in pH can alter acid–base balance in both vertebrates and invertebrates^24^, leading to for example behavioral changes in marine fish due to changes in regulation at neurotransmitters^25^ (although behavioral changes have been debated, see Clark et al.^26^). On the other hand, saturation state is the ratio of the ionic product, [Ca^2+^][CO_3_^2−^], to its saturated value. As saturation state decreases, shell development becomes increasingly constrained by kinetics and energetics^27^, although the specifics depend on the species. In marine bivalves, larval shell development and growth are dependent on seawater saturation state, and not on carbon dioxide partial pressure or pH^28^. Note that because saturation state increases slightly with temperature while pH decreases quickly with temperature, saturation states do not scale linearly with pH and each of these variables represent different aspects of ocean climate^3^.

Data for this analysis was created by combining a recent observational carbon dioxide data product, the 6th version of the Surface Ocean CO_2_ Atlas (SOCAT, 1991–2018, ~ 23 million observations), with a robust Earth System Model^29^ to provide temporal trends at individual locations of the global ocean surface for aragonite saturation state, SST, and pH from 1800–2100. Using these observation/model hybrid ensembles, we calculated the degree of global novelty or disappearance^30^ among the pre-industrial early nineteenth century (reconstructed), the late twentieth century, and twenty-first century projections under different emissions scenarios. We compared the nineteenth century pre-industrial reconstructed climate to the late twentieth century climate, and the late twentieth century climate to the late twenty-first century climate for emissions scenarios RCP 4.5 (“stabilization” emission response scenario where emissions peak in 2050, followed by slowed increase) and RCP 8.5 (worst case “business as usual” scenario where emissions peak in 2100, followed by slowed increase). Over a decade of CO_2_ emissions since 2005 show that the RCP 2.6 scenario is too low to adequately represent the future atmosphere CO_2_ level^31–33^. Consequently, the RCP 4.5 and RCP 8.5 scenarios are now the plausible low-end and high-end concentration pathways.

### Overview of metrics that reflect climate risk

We estimate the degree of global novelty or disappearance using the Mahalanobian dissimilarity metrics developed by Mahony et al.^30^. These metrics are an improvement over the standardized Euclidean distance^7^ because the latter is susceptible to variance inflation due to correlations in the raw variables and does not account for the effect of the number of variables on the statistical meaning of distance. Following Mahony et al.^30^, we estimated two metrics that reflect climatic risk: (i) Mahalanobis distance (*M*_*D*_) (a multivariate distance) between a single gridpoint at one point in time and its closest analog in the global baseline pool from another timepoint, and (ii) the transformation of *M*_*D*_ into a standardized metric called sigma dissimilarity (*σ*_D_) that can be interpreted as the number of standard deviations of interannual climate variability (ICV) at the focal station (see Table 1, definitions). The global climate baseline data includes climate normals from widespread geographic locations in the hemisphere of the focal station (e.g., northern or southern hemisphere) at a specific point of time. Following the framework outlined by Mahony et al.^30^, we interpret 2–4*σ*_D_ to represent a moderate degree of global novelty/disappearance (corresponding to the 95th percentile of the baseline) and greater than 4*σ*_D_ to represent an extreme degree of global novelty/disappearance (corresponding to the 99.994th percentile of the baseline) (see Table 1, definitions). As a statistical measure of the departure from historical variability, sigma dissimilarity provides an intrinsically meaningful metric of the general ecological significance of climatic dissimilarities^30^.

**Figure 1:**
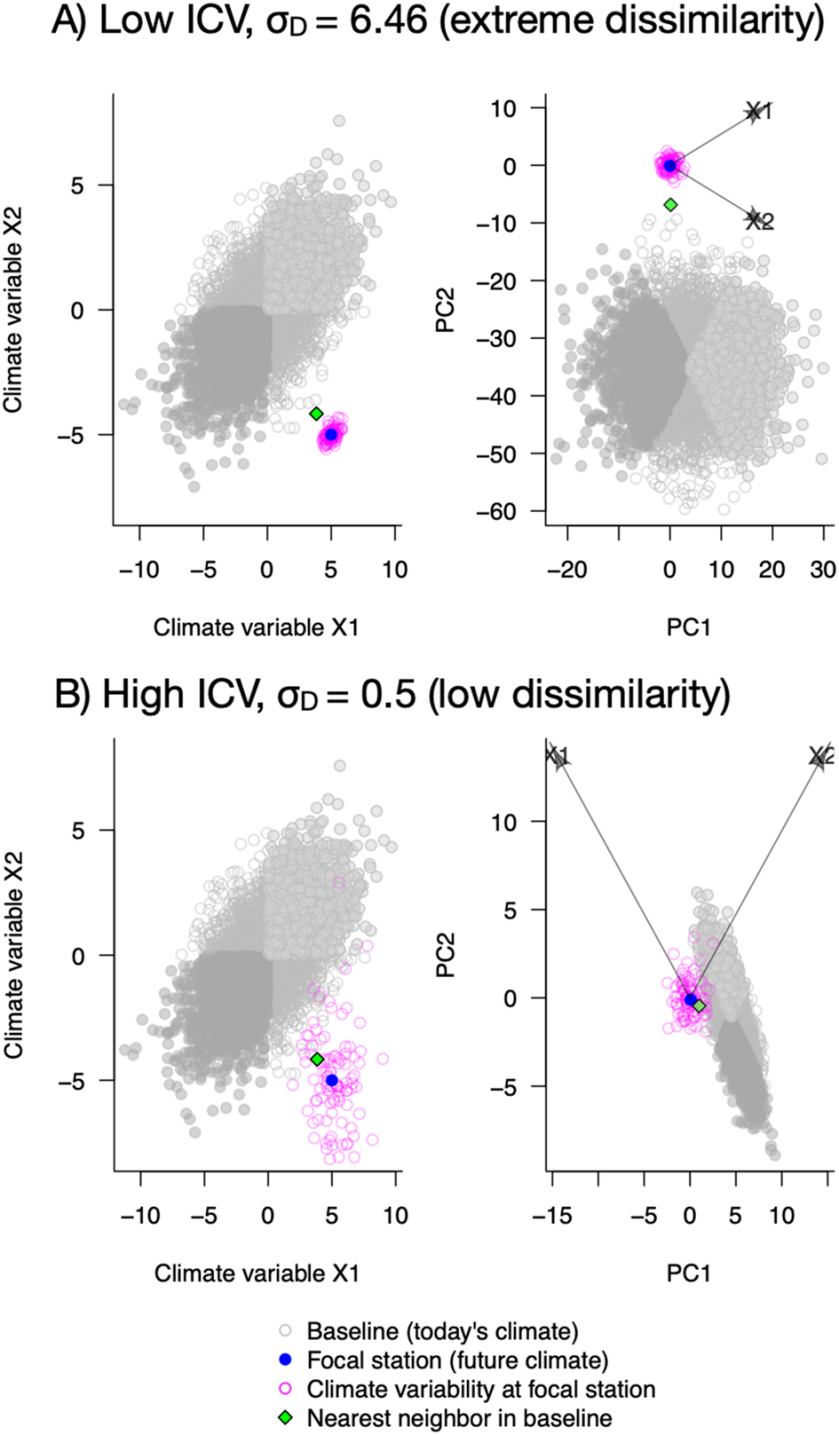
Illustration of climate novelty calculations. Hypothetical data for two focal geographic locations whose future climate normal (blue point, a novel climate in this case) is being compared to a global baseline of present-day climate normals (grey dots). The raw data (left column, for hypothetical environmental variables X1 and X2) is subject to a principal components analysis and then standardized by the multivariate interannual climate variability (ICV, pink circles) at the focal station, which results in the standardized data in the right column (arrows show how the loadings of environmental variables X1 and X2 in PC space depend on the ICV at the focal location). In the standardized PC space (right column), the degree of novelty (*σ*_D-Novelty_) is calculated as the number of standard deviations between the climate projection at the focal station (blue point) and its nearest neighbor (green diamond) in the present-day global climate baseline data (grey points, which are shaded only to help visualize the standardization). (**A**) The novelty calculation for the future climate at a focal location that experiences low ICV is calculated to be extremely dissimilar to the global baseline. (**B**) The novelty calculation for the future climate at a focal location that is projected to be the same mean future climate as A, but experiences higher ICV, is calculated to have low dissimilarity to the global baseline. Note how the different degrees of ICV for X1 and X2 affect the data transformation into PC space. The degree of disappearance (*σ*_D-Disappearance_) for a focal station is analogous to the grey points representing the global baseline for possible future climates, and the blue point representing today's climate at the focal station. For further explanation see “Overview of metrics that reflect climate risk" section in the main text.

We illustrate the calculation of sigma dissimilarity with hypothetical data in Fig. 1 for two hypothetical climate variables, X1 and X2. In the left column of Fig. 1, the grey points represent the global climate baseline data, which are shaded only for illustration. To calculate the degree of dissimilarity, *σ*_D_, a principal components analysis is performed on the global climate baseline data in the left column of Fig. 1 and standardized by the multivariate interannual climate variability (ICV, magenta circles in Fig. 1) experienced at the focal station (blue point in Fig. 1), resulting in the transformed data in the right column of Fig. 1 (see Table 1 for definitions). The different shadings of grey in the global climate data are only used to help to visualize this transformation. In this standardized principal components space, the degree of dissimilarity is then calculated as the number of standard deviations between the climate normal at the focal station (blue point in Fig. 1) and the climate normal of its nearest neighbor (e.g., closest analog) in the global climate baseline data (green diamond in Fig. 1). Via the standardization, the degree of dissimilarity calculation incorporates the amount of ICV for the focal station.

Figure 1 illustrates the calculation for the degree of global novelty for a future climate projection at a focal station compared to a present-day climate. A novel climate at a focal station occurs when a future climate normal at that location does not currently exist in the present-day baseline of climate normals from geographic locations across the same hemisphere (global climate baseline data, grey points in Fig. 1) *and* is projected to be outside that historically experienced (e.g., the ICV) at the focal station. After transformation of the raw data (Fig. 1 left column, for hypothetical environmental variables X1 and X2) with principal components and standardization by the ICV (resulting in the data in Fig. 1 right column, arrows show how the loadings of environmental variables X1 and X2 in PC space depend on the ICV), the degree of global novelty, *σ*_D-Novelty_, is an estimate of the number of standard deviations between the future climate normal at the focal station (blue point) and the climate normal of its nearest neighbor (green diamond) in the present-day global climate baseline data (grey points, which are shaded only to help visualize the standardization)^30^. In the principal components space (right side of Fig. 1), the nearest neighbor (green diamond in Fig. 1) is the geographical location in the global baseline climate data whose present-day climate normal is most similar to that of the focal station’s future projected climate normal (e.g., closest analog).

In comparing Fig. 1A,B, the future climate predicted for the focal station (blue dot) and the nearest neighbor (green diamond) is the same for both examples, but the ICV (magenta points) historically experienced at the focal station is low (in A) or high (in B). When the focal geographical location experiences low ICV, the degree of global novelty (*σ*_D-Novelty_) to its nearest neighbor is large (Fig. 1A). When the focal geographical location experiences high ICV, the degree of global novelty (*σ*_D-Novelty_) is low (Fig. 1B). Thus, when all else is equal, *σ*_D_ varies inversely with ICV. This is intuitive in the sense that a site that experiences a lot of climate variability would not be expected to be as negatively impacted by climate change as a site that experiences less climate variability.

Figure 1 can also be used to illustrate the degree of climate disappearance. A disappearing climate at a focal station is one that exists in the present-day, but is projected to no longer exist in the global baseline of future-time climates from widespread geographic locations. The degree of global disappearance (*σ*_D-Disappearance_) for a focal geographic location is analogous to the grey points representing the global baseline data for projected future climate normals and the blue point representing today's climate normal at the focal station. The magenta points still represent the ICV at the focal station, which is assumed to be constant through time. The *σ*_D-Disappearance_ is based on the number of standard deviations between the current climate normal at the focal station (blue point) and the climate normal for its nearest neighbor in the future-time climate (green diamond).

## Results

### Overview of model data

**Figure 2:**
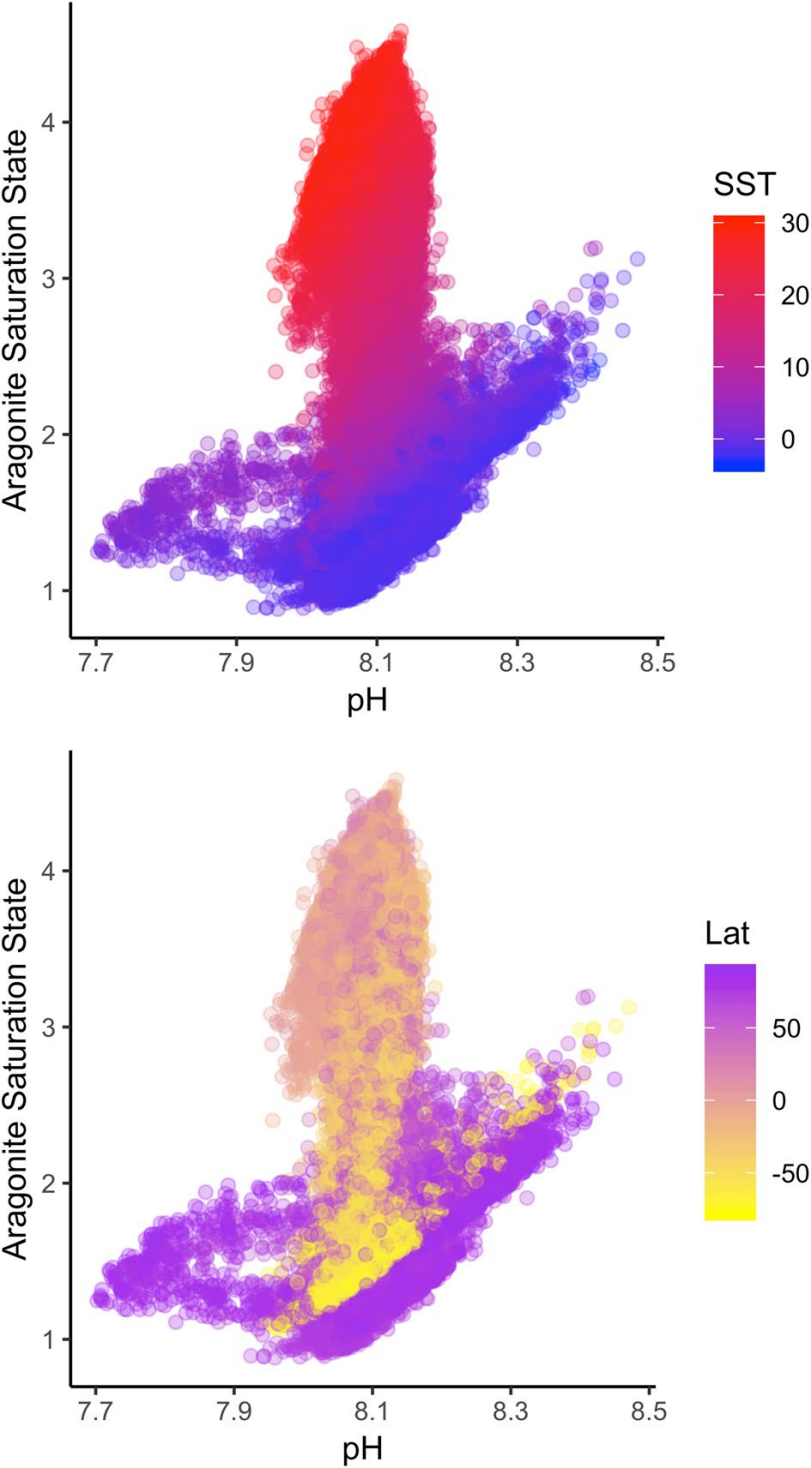
Distribution of pH versus aragonite saturation state in the global ocean. The points are colored by sea surface temperature (SST, top) or latitude (Lat, bottom).

**Figure 3:**
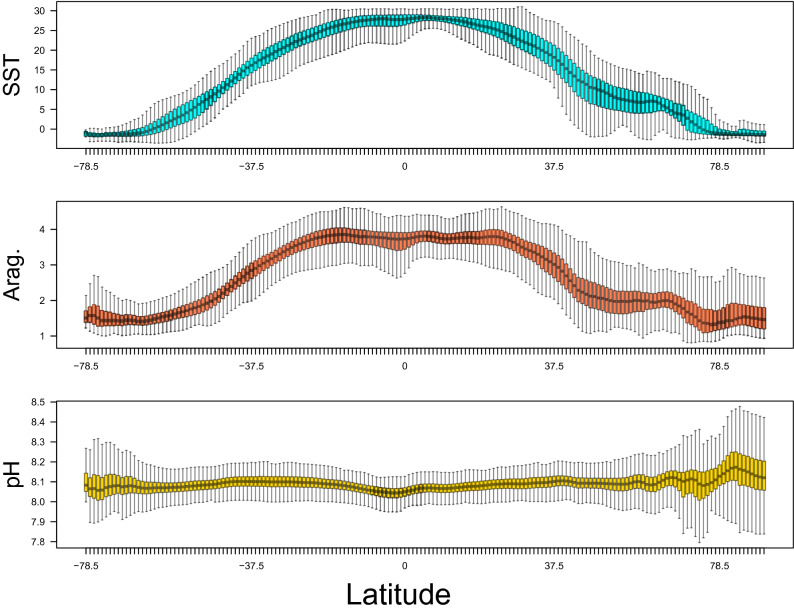
Interannual climate variability as a function of latitude. Boxplots of the interannual climate variability (ICV) used for the degree of climate novelty/disappearance as a function of latitude. The green area represents the 0.25 and 0.75 quantiles (outliers were excluded). *SST* sea surface temperature, *Arag* aragonite saturation state.

The model data has been previously published^3^, so here we only briefly summarize the patterns that are helpful in interpreting the multivariate analysis of global novelty and disappearance. The relationship between saturation state, temperature, and pH in the present-day sea surface is shown in Fig. 2. Because saturation state decreases with temperature, it does not scale linearly with pH and each of these variables represent different aspects of ocean climate. The local interannual climate variability that is used in the standardization for the degree of global novelty/disappearance calculations is typically lowest for all variables at the equator (Fig. 3). The temperate zones in the northern hemisphere typically have a more variable local ICV than temperate zones in the southern hemisphere, and the Arctic experiences lower saturation states and warmer conditions than Antarctic (Fig. 3).

**Figure 4:**
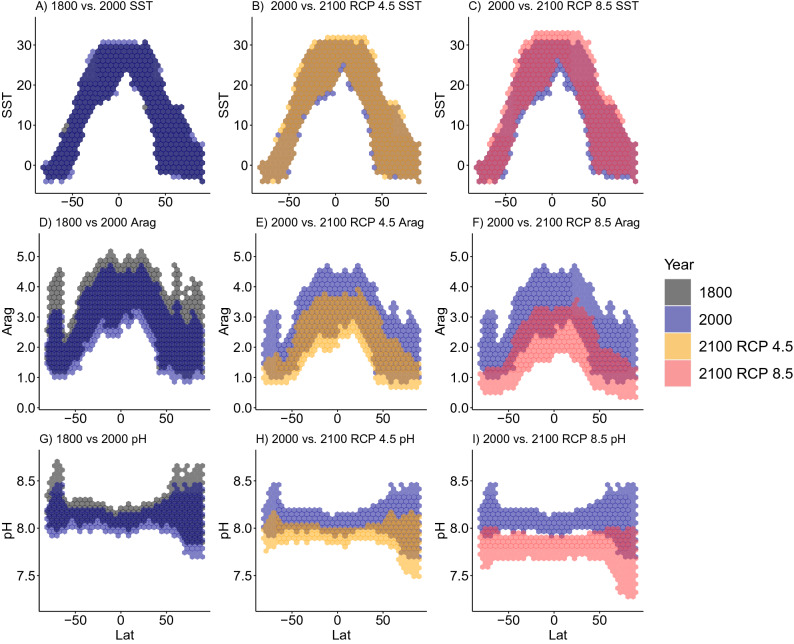
Shifts in climate variables as a function of latitude. Univariate climate change for sea surface temperature (SST, top row), aragonite saturation state (Arag, middle row), and pH (bottom row), as a function of latitude for different century comparisons. The specific comparison is described in the title of each panel.

Between 1800 and 2000, the shift in the individual climate variables as a function of latitude shows a slight temperature increase at the equator and an ocean-wide slight drop in aragonite saturation state (Fig. 4 left column). Between 2000 and 2100, these shifts are projected to become larger under RCP 4.5 (Fig. 4 middle column) and extreme under RCP 8.5 (Fig. 4 right column).

**Figure 5:**
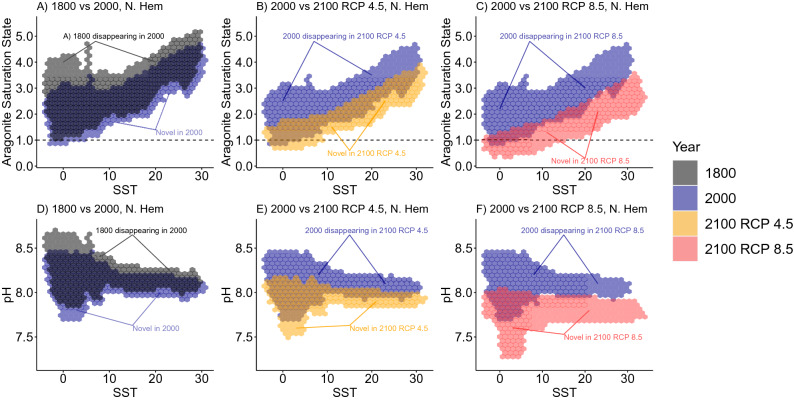
Shifts in climate envelopes for the northern hemisphere. We compared the distribution of sea surface climate normals between different centuries in the northern hemisphere. Aragonite saturation state (top row) or pH (bottom row) are plotted against sea surface temperature (SST). For specific comparisons see the titles in each panel. When aragonite saturation state falls below 1.0 (horizontal dotted line), the calcium carbonate polymorph that some marine animals use to make their shells will dissolve into seawater.

**Figure 6:**
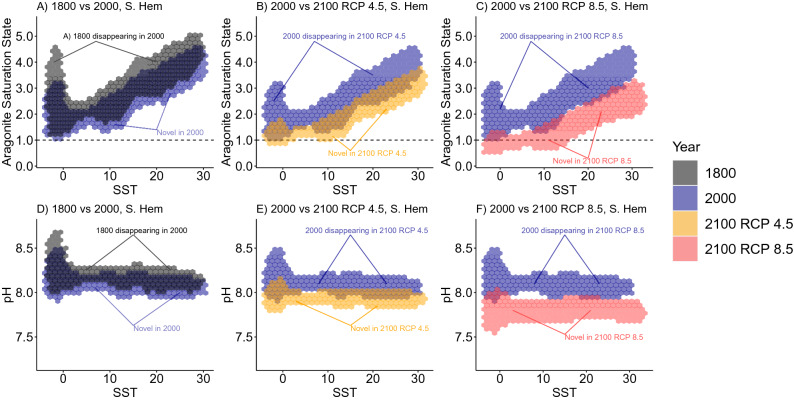
Shifts in climate envelopes for the southern hemisphere. We compared the distribution of sea surface climate normals between different centuries in the southern hemisphere. Sea surface temperature (SST) is plotted against aragonite saturation state (top row) or pH (bottom row). For specific comparisons see the titles in each panel. When aragonite saturation state falls below 1.0 (horizontal dotted line), the calcium carbonate polymorph that some marine animals use to make their shells will dissolve into seawater.

How these individual climate shifts correspond to the multivariate emergence of novel and disappearing climates is visualized in Fig. 5 (for the northern hemisphere) and Fig. 6 (for the southern hemisphere). In the northern hemisphere, the present-day undersaturated and low pH conditions in the Arctic are projected to become more common at temperate latitudes under RCP 4.5 and RCP 8.5; note that for temperate latitudes these conditions are unlikely to be globally novel because they are already common in the Arctic (Fig. 5 middle and right columns, note overlap in 2000 and 2100 envelopes at low SST). In the southern hemisphere under RCP 8.5 projections, there is almost no overlap between current and projected climate envelopes across all latitudes (Fig. 6 right column). While these figures are useful for comparing climate envelopes, note that they do not give much insight into the degree of global novelty for a specific location, because that degree depends on the amount of historical ICV at that location.

### Degree of global novelty and disappearance between 1800 and 2000

**Figure 7:**
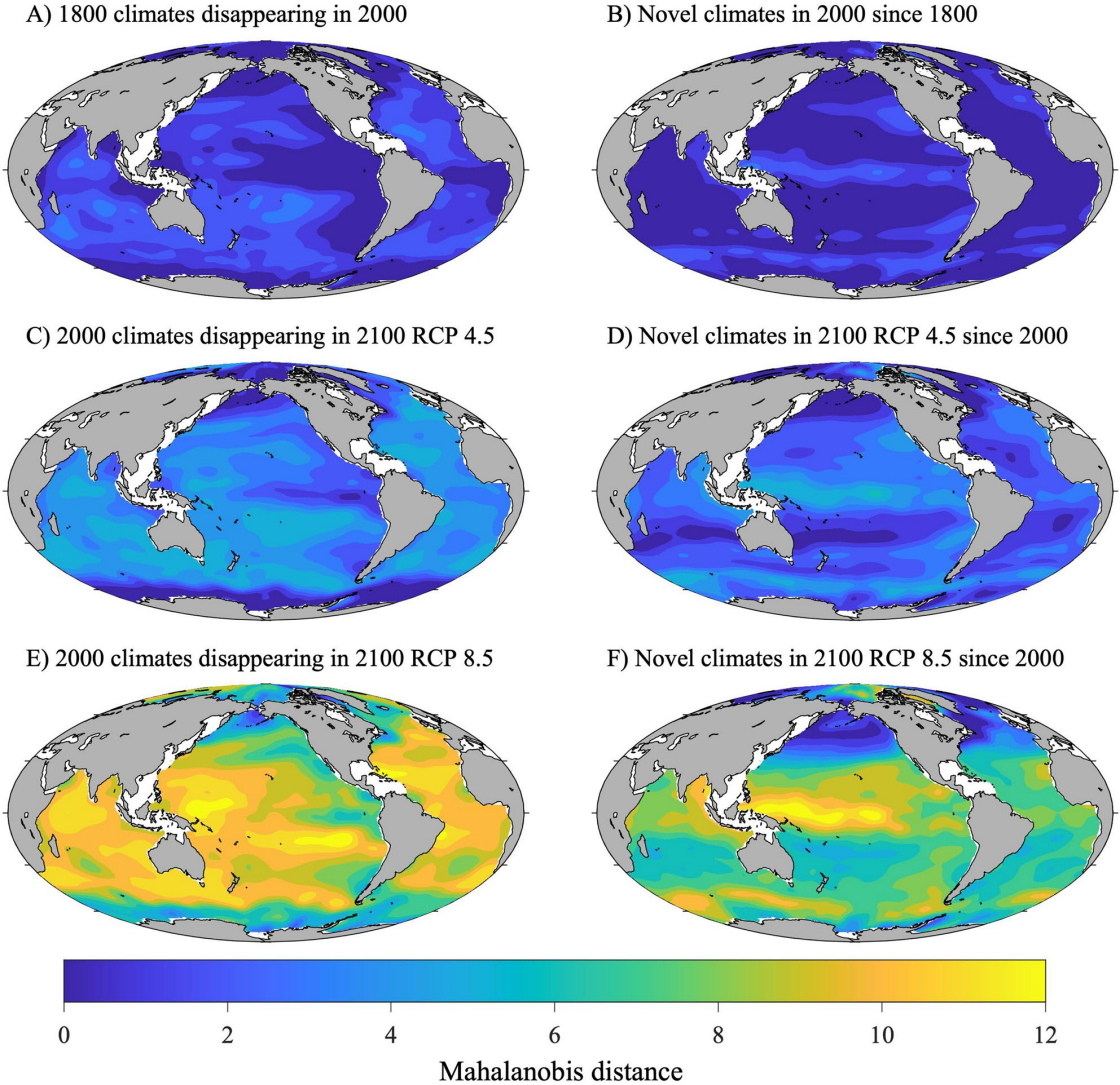
Map of climate risk based on Mahalanobis distance. A map of the multivariate distance between the climate normal for each gridpoint at one point in time and its closest analog in the global climate baseline data at another point in time (Mahalanobis distance, *M*_*D*_). The *M*_*D,Disappearanc*e_ is the multivariate distance between the climate normal of a gridpoint at an earlier time to its closest analog in the global baseline climate normals at a later time (left column). The *M*_*D,Novelty*_ is the multivariate distance between the climate normal of a gridpoint at a later time to its closest analog in the global baseline climate normals at an earlier time (right column). For specific comparisons see the titles within each panel.

**Figure 8:**
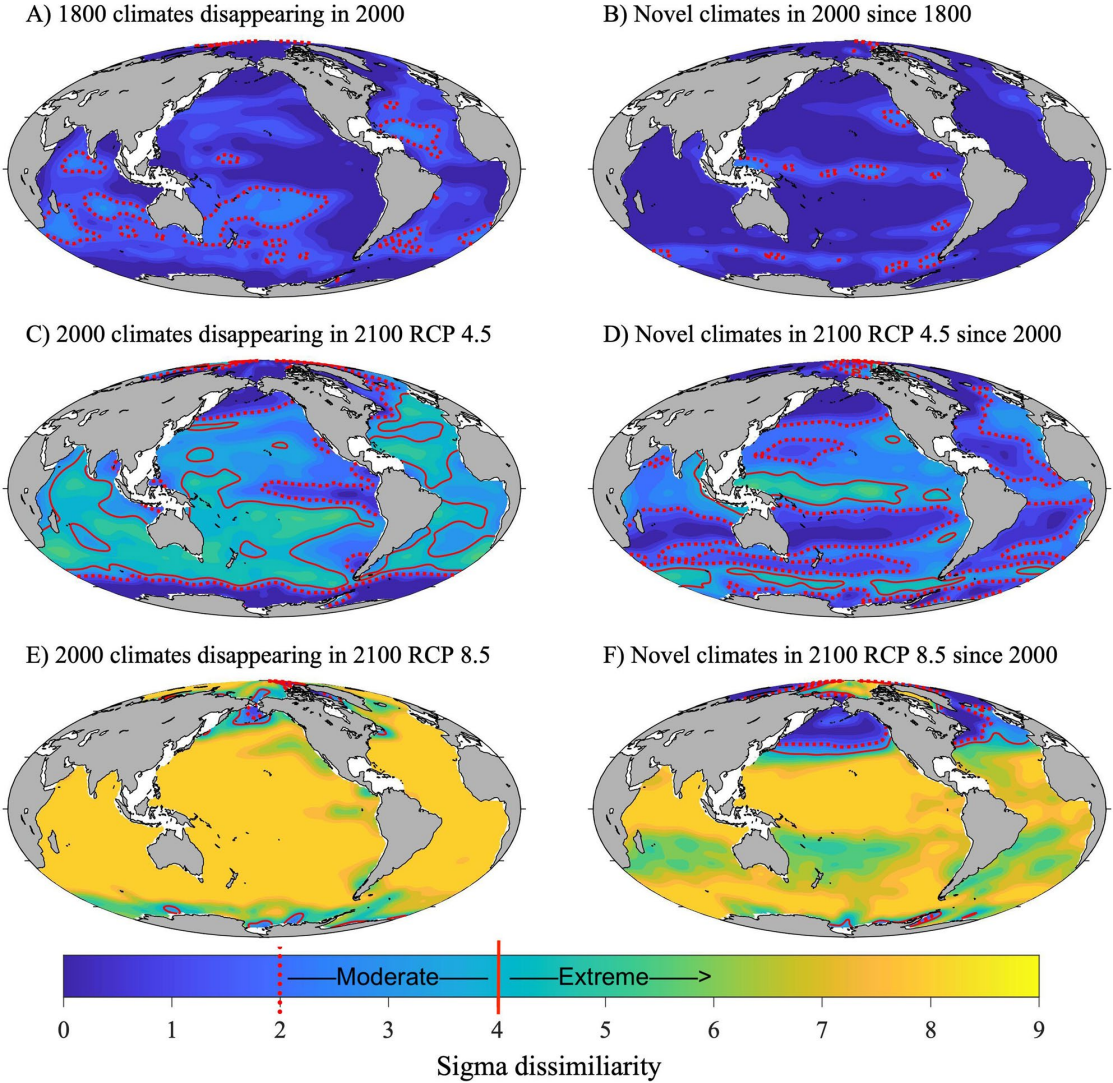
Map of climate risk based on sigma dissimilarity. The degree of global disappearance or novelty for the global ocean. Sigma dissimilarity (*σ*_D_) represents the number of standard deviations of the local interannual climatic variability (ICV) at a gridpoint at one point in time from its closest analog in a global pool of data at a different point in time. The degree of global disappearance (*σ*_D-Disappearance_) is the dissimilarity between the climate normal of a gridpoint at an earlier time to its closest analog in the global baseline climate normals at a later time (left column). The degree of global novelty (*σ*_D-Novelty_) is the dissimilarity between the climate normal of a gridpoint at a later time to its closest analog in the global baseline climate normals at an earlier time (left column). For specific comparisons see the titles within each panel. The largest *σ*_D_ that could be calculated with decimal precision was 8.29*σ*. Following^30^, a moderate degree of novelty or disappearance is given by 2 < *σ*_D_ < (corresponding to the the 95th percentile of local ICV) and an extreme degree is given by *σ*_D_ > 4 (corresponding to the the 99.994th percentile of local ICV).

Compared to 2000, 12.4% of the modeled 1800 gridpoints had moderate degree of global disappearance and 0% had an extreme degree of global disappearance (Table 2, Fig. 7A for *M*_*D-Disappearance*_ and Fig. 8A for *σ*_D*-Disappearance*_). Similarly, since 1800, 3.7% of the gridpoints from 2000 had a moderate degree of global novelty and 0% had an extreme degree of global novelty (Table 3, Fig. 7B for *M*_*D-Novelty*_ and Fig. 8B for *σ*_D*-Novelty*_). Current globally disappearing climates are trending in the Indian Ocean, the southwest Pacific, and tropical Atlantic (Fig. 7A), whereas current globally novel climates are emerging in the equatorial Pacific (Fig. 7B). The relatively small climate shift since 1800 can be visualized by the substantial overlap between the 1800 and 2000 climate envelopes for temperature and aragonite saturation state and temperature and pH (Figs. 4, 5, 6).

**Table 2:** Percent of ocean surface estimated to experience different degrees of global disappearance.

Degree of global disappearance *σ*_D-Disappearance_	1800–2000 (%)	2000–2100 RCP 4.5 (%)	2000–2100 RCP 8.5 (%)
Low (*σ*_D_ < 2)	87.6	30.2	1.5
Moderate (2 < *σ*_D_ < 4)	12.4	34.2	3.5
High (*σ*_D_ > 4)	0	35.6	95

**Table 3:** Percent of ocean surface estimated to experience different degrees of global novelty.

Degree of global novelty *σ*_D-Novelty_	1800–2000 (%)	2000–2100 RCP 4.5 (%)	2000–2100 RCP 8.5 (%)
Low (*σ*_D_ < 2)	96.3	47.6	11.4
Moderate (2 < *σ*_D_ < 4)	3.7	42.1	6.7
High (*σ*_D_ > 4)	0	10.3	81.9

### Degree of global novelty and disappearance between 2000 and 2100

A substantial proportion of the sea surface is projected to experience a moderate-to-extreme degree of global disappearance between 2000 and 2100 under RCP 4.5 and RCP 8.5. By 2100, between 35.6% (RCP 4.5) and 95% (RCP 8.5) of the sea surface is predicted to experience an extreme degree of global disappearance (Table 2, Fig. 7C,E for *M*_*D-Disappearance*_ and Fig. 8C,E for *σ*_D*-Disapperance*_). Locations with climates that are projected to experience the most extreme degree of global disappearance are primarily located in the tropics and the temperate region of the southern hemisphere (Figs. 7C,E and 8C,E), and become more widespread under RCP 8.5 (Fig. 8E).

A substantial proportion of the sea surface is also projected to experience a moderate-to-extreme degree of global novelty between 2000 and 2100 under RCP 4.5 and RCP 8.5. By 2100, between 10.3% (RCP 4.5) and 81.9% (RCP 8.5) of the sea surface is predicted to experience an extreme degree of global novelty (Table 3, Fig. 7D,F for *M*_*D-Disappearance*_ and Fig. 8D,F for *σ*_D*-Disapperance*_). Locations with climates that are projected to experience the most extreme degree of global novelty are primarily located near the equator, in the Arctic, and in the sub-polar region of the southern hemisphere (Figs. 7D,F and 8D,F), and become more widespread under RCP 8.5 (Fig. 8F).

The non-intuitive result that a larger proportion of sea surface climate will have a more extreme degree of global disappearance than degree of global novelty is caused by the way the climate envelope shifts in the northern hemisphere. A high density area of the temperature-pH envelope in 2000 does not overlap with the temperature-pH envelope in 2100 (e.g., the former would have a high degree of global disappearance). However, a high density area of the temperature-pH envelope in 2100 overlaps with some relatively rare locations in 2000 that have low temperature and low pH (thus the lower degree of global novelty). Consequently, the multivariate distance from a point at the end of the twentieth century to its nearest analog at the end of the twenty-first century (degree of global disappearance) is more often larger than the multivariate distance from a point at the end of the 21th century to its nearest analog at the end of the twentieth century (degree of global novelty).

### Comparing model ICV with real data

**Figure 9:**
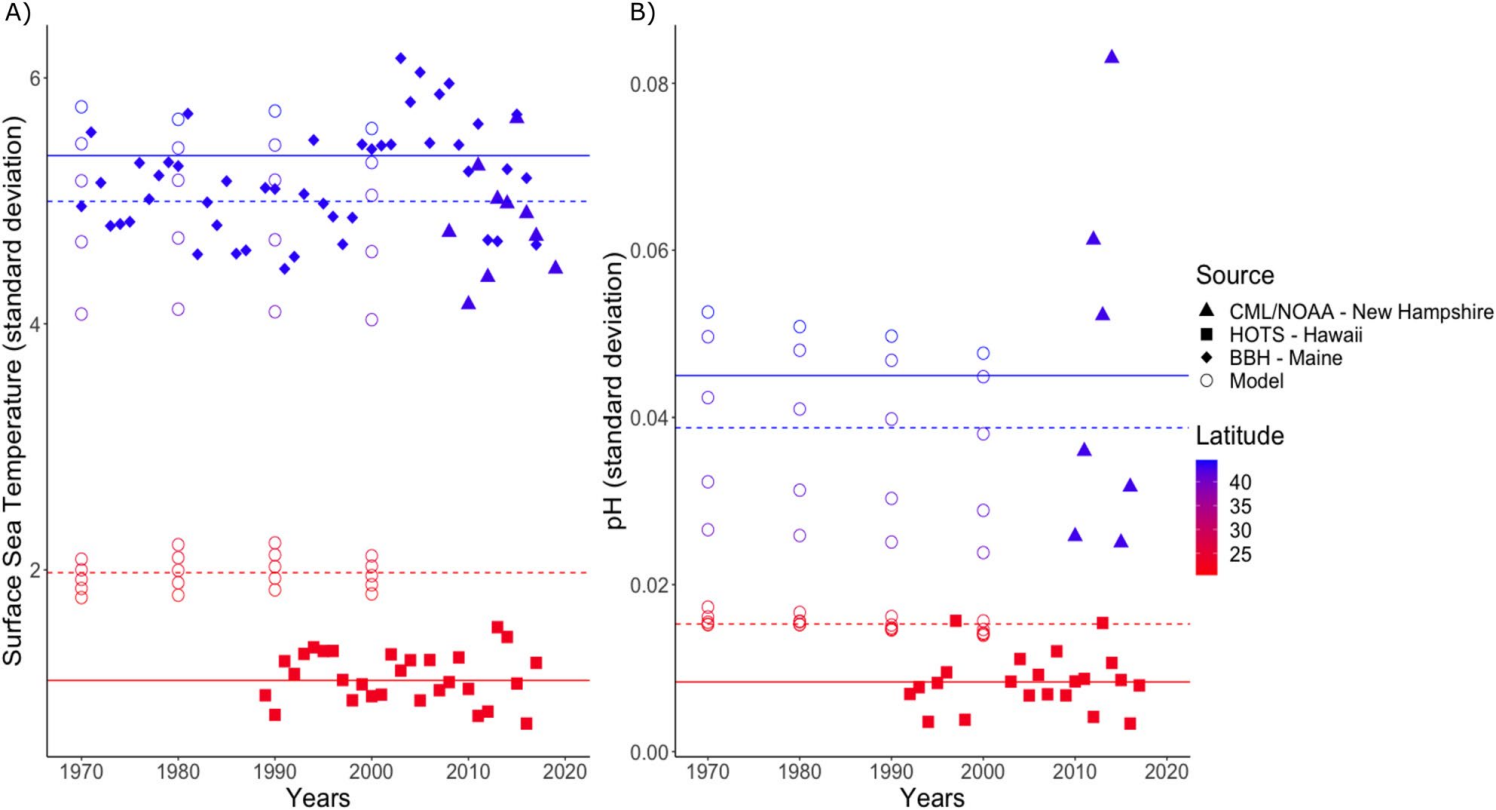
Comparison of ICV for observational versus model data. We compared long-term ocean field site measurement standard deviations in sea surface temperature (**A**) and pH (**B**) to model standard deviations at representative latitudes and longitudes. The solid lines represent the average annual standard deviation of the field measured time series data, while the dotted line represents the average standard deviation of the model in the same region. The observational data included the tropical Hawaii Ocean Time-series (HOTS), the temperate North Atlantic datasets from the University of New Hampshire Coastal Marine Laboratory and the National Oceanic and Atmospheric Administration mooring NH_70W_43N (CML/NOAA), and Boothbay Harbor, Maine (BBH).

If ICV is underestimated in our dataset, then the predictions for *M*_*D*_ and *σ*_D_ shown in Figs. 7 and 8 are overestimated. In comparing projections from the model to measured values from long-term ocean monitoring time series, we found that variation in temporal field station measurements of both SST and pH was lower in the tropics (as represented by Hawaiʻi) than at similar latitudes (between 20° N and 25° N) in the model (Fig. 9), indicating that our novelty projections for tropical regions (which are already quite large) may be underestimated. Conversely, we found that variation in field station measurements of both SST and pH was higher in the temperate zone (represented by Maine and New Hampshire) than at similar latitudes (between 40° N and 45° N) in the model (Fig. 9), indicating that our novelty projections for this region (which were among the lowest observed) may be overestimated. In summary, the qualitative prediction that equatorial regions will experience an extreme degree of global novelty and northern temperate regions will not experience globally novel conditions is robust given the direction of the slight biases in the ICV.

## Discussion

Our analysis did not predict that any modeled gridpoints on the ocean surface have experienced an extreme degree of global disappearance or novelty between 1800 and 2000. However, between 2000 and 2100 under Representative Concentrations Pathway (RCP 4.5 or 8.5) projections, our analysis predicted that a substantial proportion of the sea surface may experience an extreme degree of global novelty and disappearance relative to the global climate baseline data. The upward estimates in our analysis are larger than those projected for global novel and disappearing climates on land^7^, and are due in part to the ocean surface environment being two to three orders of magnitude less variable than that on land^34^. Under both RCP 4.5 and RCP 8.5, the more extreme degree of global novelty near the equator and in the sub-Antarctic is driven in part by the lower interannual climatic variability (ICV) at these locations. In contrast, the low degree of global novelty in northern temperate regions stems in part from the higher ICV at those latitudes.

In this study we estimated the degree of multivariate novelty of future climates and disappearance of extant climates relative to global climate baseline data for the sea surface, which complements previous studies for the global ocean based on local rates of climate change^15,16^. The local versus global metrics provide important, and different, information about the vulnerability of populations to climate change. Local climate change at a specific location, relative to the historical variability at that location, may reflect the extent to which the species composition will shift as species track shifting climate envelopes with dispersal. The degree of global climate novelty at a location, however, may indicate how stressful novel conditions will be for all species. In contrast, the degree of global climate disappearance for a location may represent how hard it might be for species who are well adapted to the climate at that location to find a similar climate in the future.

While dispersal limitations greatly increase the risk that species will experience the loss of extant climates or the occurrence of novel climates^7^, the high dispersal potential of marine organisms with a planktonic larval stage has been discussed as a trait that will allow them to keep pace with climate change^15^. Recent studies have found that marine species are able to track shifting climates^10,11^. Our study shows that the majority of these climate shifts are not novel (e.g., have an analog) since the early nineteenth century. In other words, although some climate variables, such as pH, have already emerged beyond historical baseline for a particular location^15^, our study shows these climates are not novel from a global perspective and may facilitate tracking via range shifts. However, if a majority of the ocean surface climate disappears and is replaced by novel climates with no recent analog by the end of the twenty-first century, the optimal environment for many species may not exist and dispersal will not help these species keep pace with environmental change. Instead, species may need to keep pace via evolutionary adaptation, plasticity and acclimatization, and/or epigenetic processes^35^.

Evidence for adaptive capacity is emerging, although examples are still few. Phytoplankton have been shown to evolve rapidly in response to increased *p*CO_2_^13^, due in part to their short generation times and large population sizes. The concerns remain, however, that adaptive variation for high *p*CO_2_ is limited in most species^36^, that high *p*CO_2_ can diminish the heritability of larval traits^37^, that marine species live close to their upper thermal limits^38^, and that the unprecedented rate of change will be too fast relative to the long life span of many marine species for adaptive evolution to occur before their lineages go extinct^39^. Yet, a growing number of studies have found transgenerational plasticity of marine invertebrates and vertebrates in response to increased temperature or *p*CO_2_^40,41^, suggesting that non-genetic or epigenetic processes could play a major role in acclimatization—although there are many knowledge gaps in the molecular mechanisms that underlie such processes^42^.

The degree of global novelty or disappearance for a specific location is relative to the amount of variability historically experienced at a location. We found that the model data tended to have lower ICV than time series data for high latitudes, and higher ICV than time series data for low latitudes, indicating that our estimates of novelty may be overestimated for high latitudes (which are already the lowest novelty) and underestimated for low latitudes (which are already the highest novelty). Therefore, the main conclusion—that the equator and sub-Antarctic regions will experience the highest degree of global novelty and northern temperate regions the lowest degree of global novelty—is robust to the direction of bias we observed in the ICV. Note, however, that our analysis did not include coastal areas, which are known to experience large fluctuations in temperature and carbonate chemistry due to upwelling processes and freshwater input^43,44^. Including coastal areas in this analysis was not possible due to the paucity of data, but would be an important avenue for future research.

Our projections may be conservative because there are other important aspects of seawater chemistry, food availability, and ocean dynamics that will be altered by climate change but were not considered by our model. For example, enhanced stratification caused by warming temperatures can have a range of indirect effects, including reduced nutrient supply to phytoplankton at low latitudes, but a more favorable light regime for these organisms at high latitudes^45,46^. Primary productivity may also be altered in coastal areas where productivity is driven by the seasonal upwelling of deep, nutrient-rich water. Climate change is altering the intensity, timing and spatial structure of upwelling dynamics, thus reshaping patterns of primary productivity^47–50^. Warming also reduces the solubility of oxygen, and hypoxic conditions have been shown to have negative effects on many marine organisms^51^. Moreover, warming drives sea ice melt and systematic freshening of polar areas^52^.

Including multiple stressors into calculations of *M*_*D*_ and *σ*_D_ is an important avenue for future research. In our analysis, reconstructed and projected carbonate chemistry for the global ocean was based on the GFDL-ESM2M model that is often considered as the most reliable model for the carbonate parameters (Dunne et al. 2012, 2013). Other models with different variables (e.g. sea ice, salinity, dissolved oxygen, nutrients, etc.) could be analysed in the same way and this would allow an estimate of the uncertainty in the results. The sensitivity of the results to the choice of model is an important next step towards producing more robust estimates of novelty and disappearance.

If the projections of climate novelty and disappearance reported here are accurate, the cascading effects on marine ecosystems and communities could be substantial. Areas such as the IndoPacific, which are projected to experience the most extreme degree of climate novelty and disappearance, are critical hot spots for endemic biodiversity and coral reefs^53–55^. Coral reefs are particularly vulnerable to bleaching of their zooxanthellae symbionts, which can result from minor increases in temperature^50^. In these areas, elevated risks of ecological surprises, including extinction, are likely.

Shifting climate niches only represent one aspect of the ecological risks associated with climate change. Modified energy flows and biogeochemical cycles, multiple stressors, shifts in phenology, climate-mediated invasions, climate-driven disease outbreaks, and asynchronies between prey availability and predator demand are some of the other processes that will contribute to shifting ecosystem distributions and the services that they provide to society^50,56,57^. Species will vary in their ability to keep up with multivariate environmental transitions into no-analog climates, which will promote the formation of no-analog species assemblages and present many ecological surprises. Highly novel marine ecosystems will challenge the predictive ability of eco-evolutionary models and present many challenges to the preservation of marine biodiversity over the next century.

## Methods

### Global ocean reconstructed and projected data

Seawater carbonate data for pH and aragonite saturation state calculation in this study were extracted from the 6th version of the Surface Ocean CO2 Atlas (SOCATv6, 1991–2018, ~ 23 million observations) at a spatial resolution of 1 × 1 degree^58^. Data without quality control flags of A or B (uncertainty of fugacity of carbon dioxide, fCO_2_ < 2 µatm) were omitted. Silicate and phosphate values for all SOCATv6 stations were extracted from the gridded GLODAPv2 climatologies^59^. Total alkalinity (TA) was then calculated with the updated Locally Interpolated Alkalinity Regression (LIARv2) method^60^. pH on the total hydrogen scale (pH_T_) and aragonite saturation state were calculated from in-situ temperature, salinity, hydrostatic pressure, dissolved inorganic carbon (DIC) concentration, TA, silicate and phosphate. Dissociation constants were taken from the literature for carbonic acid^61^, bisulfate (HSO_4_^−^)^62^, and hydrofluoric acid (HF)^63^. Total borate concentration equations were the same as reported by Uppström^64^. A MATLAB version^65^ of the CO2SYS program^66^ was used for analysis. Uncertainties of the methods using the CO2SYS errors program^67^ are estimated to be 0.01 for pH and 0.13 for aragonite saturation state, assuming uncertainties for SST, salinity, TA, and DIC of 0.01, 0.02, 6 µmol kg^−1^ and 4 µmol kg^−1^, respectively.

The calculated pH_T_ and aragonite were then adjusted from their sampling year to 2000 assuming that: (a) sea surface *p*CO_2_ increases at the same rate as atmospheric mole fraction of carbon dioxide (xCO_2_), as documented by the IPCC Fifth Assessment Report 5 (AR5)^68^, (b) SST increases at the rate described by NOAA’s Extended Reconstructed Sea Surface Temperature (ERSST) v5^69^, and (c) salinity and TA remain constant. Surface pH_T_ and Revelle Factor were further adjusted from their sampling month to all 12 months of 2000 assuming that: (a) sea surface *p*CO_2_ follows the same annual cycle as documented by the LDEO database^70^, (b) sea surface temperature (SST) in all months of 2000 can be approximated by the 1995–2004 average monthly SST climatology from the World Ocean Atlas^71^ and (c) salinity and TA remain constant.

Surface ocean pH_T_ and aragonite saturation state in all 12 months for all decades from 1770 to 2100 under the IPCC scenarios (RCP 4.5 and RCP 8.5) were reconstructed or projected assuming that sea surface *p*CO_2_ and SST increase at the rate simulated by the GFDL-ESM2M model run with these pathways^2,29^. Spatial mapping was conducted using a Matlab version (Divand Software) of the Data-Interpolating Variational Analysis (DIVA)^72^. For more detail, please refer to Jiang et al.^3^.

### Estimating global climate novelty or disappearance

For each location, the metrics we estimate are based on dissimilarity between the projected multivariate (past or future/projected) climate change at a given location and its nearest analog in a global set of “baseline” data. To estimate the range of possible degrees of novelty or disappearance, we compared the predictions for a global baseline and a hemisphere-restricted baseline (e.g., northern or southern hemisphere). An important feature of our calculations is that they (i) are performed in multivariate space, and (ii) take into account the interannual climatic variability (ICV) at that location. For all analyses, climate normals were calculated based on 40-year means of each climate variable and ICV was based on model data for 1965–2004 (see Table 1 for definitions). Because the ICV did not change substantially through time in the model data, our results were not sensitive to the span of years chosen to represent the ICV.

Aragonite saturation state was log_10_-transformed because it was a ratio variable: it is limited at zero and proportional changes are meaningful (C. R. Mahoney, *pers. comm.*). This transformation makes the difference between 1 and 2 the same significance as the difference between 0.5 and 1 (doubling vs. halving, i.e., proportional scaling). In practice, temperature doesn’t need to be log-transformed because it doesn’t vary across orders of magnitude, and in our case pH is already a log-scaled variable. Nevertheless, our analysis was not sensitive to whether or not aragonite saturation state was log-transformed.

The degree of global novelty is calculated by comparing a *later* climate normal for *each* ocean gridpoint to all *earlier* climate normals for the global baseline data. We performed three planned comparisons for the degree of climate novelty as measured by *M*_*D-novelty*_ and *σ*_D*-novelty*_ (note we use the reference period 1965–2004 ICV for all analyses): (i) novelty of the late twentieth century ocean surface (1965–2004) compared to pre-industrial early nineteenth century (1795–1834) reconstructed climate; (ii) novelty of the late twenty-first century climate under RCP 4.5 (2065–2104) compared to late twentieth century; and (iii) novelty of the late twenty-first century climate under RCP 8.5 (2065–2104) compared to late twentieth century.

Conversely, the degree of global disappearance is calculated by comparing an *earlier* climate normal for *each* ocean gridpoint to a *later* pool of climate normals for the global baseline data. High values indicate places where climates may disappear; i.e., they have no close counterpart anywhere in the later timepoint. We performed three planned comparisons for the degree of climate disappearance as measured by *M*_*D-disapperance*_ and *σ*_D*-disappearance*_: (i) disappearance of climates from early nineteenth century pre-industrial times (1795–1835 reconstructed climate) in the late twentieth century ocean (1965–2005); (ii) disappearance of late twentieth century climates by the late twenty-first century under RCP 4.5 (2065–2105); and (iii) disappearance of twentieth century climates by the late twenty-first century under RCP 8.5 (2065–2105).

Values of *σ*_D_ higher than ~ 8 were difficult to estimate due to the high decimal precision required to estimate probability in the extreme tail of the chi distribution; in these cases *σ*_D_ was set to a value of 8.29 *σ* (the maximum value that could be calculated given decimal precision). We created maps of *M*_*D*_ and *σ*_D_ in Matlab R2021 Version (code available in repo).

### Comparing ICV between model projections and real data

Because of the sensitivity of the degree of global novelty/disappearance calculations to the ICV (see *An overview of global climate novelty and disappearance calculations*), we wanted to ensure the ICV that we used from the model data were similar to those observed in long-term ocean time series. We were particularly concerned whether ICV might be underestimated in the model data, because that would bias our estimates of M_D_ and *σ*_D_ upwards. To explore whether ICV in the model projections were lower or greater than that observed in long-term ocean time series, and to address some of the limitations due to the coarse grid of global models, we compared SST and pH standard deviations from the model to those from long-term ocean monitoring time series in the tropical and temperate zones.

The model output is the predicted climate variable for that ocean gridpoint for each month, and it is calculated every decade. The standard deviation of the modeled data is based on the monthly data for each year that data is available. The observational data, however, is collected continuously across an entire year. The standard deviation of the observational data is based on this continuous data for each year that data is available.

Real measurements of SST and surface pH were downloaded from Hawaii Ocean Time Series^73^, and from the University of New Hampshire Coastal Marine Laboratory^74^; SST was also acquired from Boothbay Harbor^75^; and additional pH measurements from the National Oceanic and Atmospheric Administration mooring NH_70W_43N (NOAA)^76^. For regional comparisons, the tropical central Pacific (HOTS) dataset was compared to model data for values between latitude 20° N and 25° N, and longitude 160° E and 130° W, and the temperate North Atlantic (UNH_CML, BBH & NOAA) data were compared to model data for values between latitude 40° N and 45° N, and longitudes 40° W and 70° W. For the time series data, years in which measurements were not sampled continuously across both winter and summer months were omitted. Yearly standard deviations of both SST and pH for the observational and model data were calculated and compared in R.

## Data Availability

Code and data for reproducing the results can be found at the Dryad repository: *Data from: Novel and disappearing climates in the global surface ocean from 1800 to 2100* (doi:10.5061/dryad.ht76hdrgb).
